# Transdermal drug delivery of labetalol hydrochloride: Feasibility and effect of penetration enhancers

**DOI:** 10.4103/0975-7406.72132

**Published:** 2010

**Authors:** Saqib Zafar, Asgar Ali, Mohammed Aqil, Abdul Ahad

**Affiliations:** Abbott Vascular, New Friend’s Colony Okhla, New Delhi - 110 025, India; 1 Faculty of Pharmacy, Hamdard University, New Delhi - 110 062, India

**Keywords:** Drug delivery, labetalol, penetration enhancer, skin permeation, transdermal

## Abstract

**Objectives:**

The objective of this study is to investigate the feasibility of transdermal drug delivery of Labetalol Hydrochloride (LHCl) and to study the effect of different penetration enhancers on the skin permeability of LHCl.

**Methods:**

The permeability experiments were conducted using a horizontal glass diffusion cell with a diffusional area of 2.37 cm-^2^ on albino rat skin. The effect of various penetration enhancers namely turpentine oil, dimethyl formamide (DMF), menthol, dimethyl sulfoxide, pine oil, and 2-pyrollidone, and the effect of the concentration of drug and enhancer in the donor phase on the skin permeability of LHCl was studied.

**Results:**

The apparent partition coefficient of the drug was found to be 6.95, suggesting it to be a lipophilic drug. The preliminary skin permeation studies revealed that the permeation of LHCL through albino rat skin was moderate (K_p_ = 6.490 × 10^-2^ cm hr^-1^) from isotonic phosphate buffer of pH 7.4. An appreciable increase in the LHCl permeability coefficient was observed on using a co-solvent (ethanol 95%) with the penetration enhancers in the donor phase. DMSO (10% v/v) was found to be the most effective enhancer for Labetalol hydrochloride (Enhancement Factor = 1.165). An increase in the concentration of drug and enhancer in the donor cell accentuated the permeability coefficient of LHCl.

**Conclusions:**

It was concluded that LHCl could be delivered via the dermal route with the use of 10% DMSO as the penetration enhancer.

Conventional drug therapy, although most popular and widely used, is not without its deficiencies and limitations. For most therapies the plasma and blood levels of the active constituents is not maintained for a long period of time and hence frequent dosing is required. This reduces patient compliance. Second, most of the drug is bio-transformed or reduced to inactive forms by hepatic and other enzymes in the body. This is particularly true for oral formulations. To overcome this effect parenteral forms are available, but again they are very expensive, have a short shelf life and are painful to administer. These factors, along with the intrinsic inability of conventional dosage forms to achieve spatial placement and specific targeting, is a compelling motive for investigation of controlled release drug delivery systems.

As they provide a steady plasma level in the therapeutic range for a long time, transdermal systems are particularly useful when drug therapy is required for chronic ailments or for a long period of time.[1] Therefore the formulation of a transdermal system for hypertension is a rational prospect.

Hypertension refers to an increase in pressure in any blood vessel, such as pulmonary or portal hypertension. It usually refers to elevated systemic arterial blood pressure. Hypertension is a not disease, but a physical finding, and expert opinion supports and accepts hypertension as a diastolic pressure of more than 90 torr, as at this value the frequency of complications due to hypertension rise significantly. A number of antihypertensive drugs are used for the treatment of hypertension and the most prominent among them are the adrenergic α and β blockers. The β blockade reduces the cardiovascular output by decreasing the heart rate through β_2_ receptors in the heart, whereas, the a blockade decreases myocardial workload by causing vasodilatation of vessels through the α_1_ receptors in the blood vessels.

Transdermal therapy is usually restricted to a limited number of drugs, as the stratum corneum presents a formidable barrier toward permeating substances.[2] However, attempts have been made to alter the permeability of the stratum corneum by the use of penetration facilitators,[3–15] for achieving better permeation of the drugs.

Labetalol hydrochloride (LHCl) is an antihypertensive drug belonging to the class of β blockers. It has a low biological half-life of 2-5 hours and undergoes extensive pre-systemic metabolism ranging from 14 – 89%.[16,17] It has a low molecular weight (364.9), with no reported skin irritation history. It also has a favorable partition coefficient (7.08). With all these characteristics we propose LHCl to be an ideal drug candidate for the development of a transdermal therapeutic system (TTS).

The present study is an attempt to explore the feasibility of transdermal drug delivery of LHCL, and to study the influence of various penetration promoters on the permeability of the drug through rat skin. There are no previous reports on the skin permeation of labetalol.

## Materials and Methods

### Materials

LHCL was a kind gift from Neuland Laboratories, India. Turpentine oil, pine oil, 2 pyrrolidone, dimethyl sulfoxide (DMSO), dimethylformamide (DMF), and menthol were purchased from S.D Fine Chemicals (India). All other chemicals were of AR grade and were used as received.

### Animals

Male albino rats (8 weeks old, 200 – 250 g) were supplied by the Central Animal House of Hamdard University and inhabited under standard laboratory conditions in 12 hours light / dark cycle at 25 ± 2 °C. The animals were nourished with a pellet diet (Lipton, India) and water *ad libitum*. The animals were received after the study was duly approved by the University Animal Ethics Committee.

### Analytical method

Ten milligrams of LHCl was accurately weighed using an electronic balance and was dissolved in 50 ml of ethanolic IPB of pH 7.4 (1:9) and 5 ml of 0.1 M HCl was added. It was diluted up to 100 ml with ethanolic IPB of pH 7.4 (1:9). From this, 50 ml was taken and diluted with ethanolic IPB of pH 7.4: 0.1 M HCl (95:5), to prepare a stock solution of 50 *μ*g/ml. Serial dilutions were prepared using different drug concentrations from 2 to 24 *μ*g/ml using ethanolic IPB of pH 7.4: 0.1 M HCl (95:5). Absorbance of these dilutions was taken using a double beam UV spectrophotometer (Shimadzu, Japan) at λ_max_ = 302 nm, using ethanolic IPB of pH 7.4: 0.1 M HCl (95:5) solution as the blank. A graph was plotted between concentration (abscissa) and absorbance (ordinate). The graph obeyed the Beer–Lambert law in the selected concentration range with a correlation coefficient of 0.9981.

### Determination of the apparent partition coefficient

The apparent partition coefficient (APC) of LHCl was determined in n-octanol / IPB of pH 7.4. A solution of 20 *μ*g/ml of LHCl was prepared in a phosphate buffer and 20 ml of the solution was then transferred to a calibrated tube (50 ml capacity) containing 20 ml of n-octanol. The tube was sealed with a stopper and then agitated for 24 hours in a thermostatic water bath maintained at 37 ± 0.5 ° C, for one hour. The two phases were then separated using a separating funnel and the drug concentration was analyzed in the buffer phase at λ_max_ 302 nm using a double beam spectrophotometer (Shimadzu, Japan). The drug concentration in the n-octanol phase was determined as the difference between the initial and final concentration of the drug in the buffer phase.

The APC of LHCl was determined by the following formula:

$$\mathrm{APC}\;=\;C_{o}/C_{b}$$

Where Co is the concentration of drug in the n-octanol and C_b_ is the concentration of drug in the buffer.

### Skin permeation experiments

A modified horizontal glass diffusion cell[18] was used for the preliminary screening of drug permeation across the albino rat skin. The cell consisted of two half cells, the donor and the receiver, which were held together with springs. The area of diffusion between the two half-cells was 2.37 cm^2^. The capacity of each half-cell was 10 ml. The albino rats were sacrificed with prolonged ether anesthesia. The skin was excised from the abdominal region and dermatomed prior to storage at −40 ° C until further use. On the day of the experiment the skin was thawed to room temperature and the hair and fat were removed. The skin was washed with distilled water and was trimmed to the appropriate size and mounted on the diffusion cell such that the stratum corneum of the skin faced the donor cell and the dermis faced the receiver cell. The cell was mounted using springs. The receptor compartment was filled with the vehicle (i.e., IPB pH 7.4), containing 0.003% w/v sodium azide as a preservative and the donor compartment was filled with drug solution (100 ug/ ml) in different study vehicles, with or without penetration enhancers [Table 1].

**Table 1:** Study design of preliminary skin permeation of LHCl through rat skin

Study code	Vehicle in donor compartment	Vehicle in receptor compartment	Penetration enhancer concentration (% w/v)	
PS1	Distilled water	IPB of pH 7.4	None	
PS2	IPB of pH 7.4	IPB of pH 7.4	None	
PS3	Distilled water	IPB of pH 7.4	aDMF (5%)	
PS4	Distilled water	IPB of pH 7.4	bDMSO (5%)	
PS5	Distilled water	IPB of pH 7.4	2-Pyrrolidone (5%)	
PS6	Ethanolic IPB of pH 7.4 (1:9)	IPB of pH 7.4	None	
PS7	Ethanolic IPB of pH 7.4 (1:9)	IPB of pH 7.4	Turpentine Oil (5%)	
PS8	Ethanolic IPB of pH 7.4 (1:9)	IPB of pH 7.4	Pine Oil (5%)	
PS9	Ethanolic IPB of pH 7.4 (1:9)	IPB of pH 7.4	Menthol (5%)	
PS10*	Distilled water	IPB of pH 7.4	None	
PS11*	IPB of pH 7.4	IPB of pH 7.4	None	
PS12	Distilled water	IPB of pH 7.4	DMSO (7.5%)	
PS13	Distilled water	IPB of pH 7.4	DMSO (10%)	

* Drug concentration in donor compartment = 200 *μ*g/ml

a DMF = Dimethyl Formamide

b DMSO = Dimethyl Sulfoxide. Drug concentration in donor compartment = 100 *μ*g/ml

**Figure 1:**
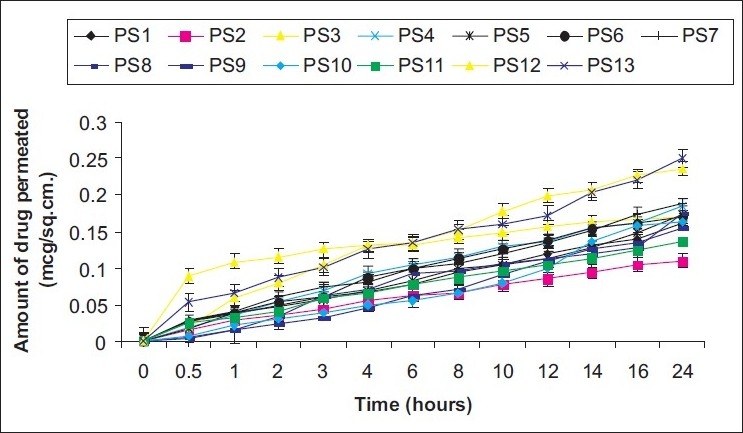
Permeation profile of labetalol hydrochloride through rat skin from different donor solutions

The aliquots (1 ml) of the receiver phase were withdrawn at intervals of 0, 0.5, 1, 2, 3, 4, 6, 8, 10, 12, 14, 16, and 24 hours, filtered and analyzed for drug content by spectrophotometry at 302 nm. Graphs [Figure 1] were plotted between cumulative amount of drug permeated (*μ*g/cm^2^) versus time (hours). The permeability coefficient (K_p_) was calculated as the quotient of flux and drug donor concentration. The enhancement factor (EF) was calculated as the ratio of K_p_ with enhancer and K_p_ without enhancer (control), to ascertain the effect of the various enhancers.[19] The influence of various vehicles, drug concentration, and enhancer concentration was also studied.

## Results and Discussion

The apparent partition coefficient of LHCl was found to be 6.95, which correlated well with the reported value of 7.08.[17] The result suggested that LHCL was a suitable candidate for transdermal drug delivery, as it was sufficiently lipophilic, with the desired water solubility (1 in 30), which would allow penetration through the hydrophilic pores in addition to drug diffusion through the lipoprotein barrier of the stratum corneum by virtue of drug lipophilicity.

The preliminary *in-vitro* skin permeation studies revealed that LHCl permeation through rat skin in distilled water was appreciable (Kp = 6.322 × 10^−2^cm^2^hr^−1^). A significant difference (*P* < 0.05) was seen between the skin permeation rates obtained from distilled water solution and those from isotonic phosphate buffer of pH 7.4. Addition of a co-solvent (ethanol 95%) resulted in an insignificant increase (*P* > 0.05) in the permeability coefficient [Table 2]. Different penetration enhancers were tried in an attempt to augment the permeation of LHCl. Initially, all the enhancers (turpentine oil, dimethyl formamide (DMF), menthol, dimethyl sulfoxide, pine oil and 2-pyrollidone) were used in 5% v/v concentration. All the treatments increased the permeability coefficient of the drug with respect to the respective controls. The co-solvent, ethanol 95%, was used along with IPB of pH 7.4, in a ratio of 1:9, for enhancers that were not soluble in the isotonic phosphate buffer. Therefore, for DMSO, DMF, and 2-pyrrolidone, the control was distilled water and for turpentine oil, menthol, and pine oil, ethanolic IPB of pH 7.4 was taken as control. The effectiveness of these penetration enhancers was ascertained by the determination of the enhancement factor (EF) of each enhancer. The EF of DMSO was found to be higher than those of turpentine oil, DMF, pine oil, 2-pyrrolidone, and menthol [Table 3]. The increase in the permeability coefficient with 5% DMSO was close to 17% and it was found to be the most effective penetration enhancer. DMSO enhanced the skin permeation by either reversibly changing the configuration of the protein structure of the stratum corneum or by increasing the thermodynamic activity of the drug. DMSO also enhanced permeation by causing a swelling in the stratum corneum to induce formation of channels, which decreased diffusional resistance.[12]

**Table 2:** The effect of various drug solutions on the skin permeability of LHCl

Study code	Drug solution	*Permeability coefficient K_p_× 10^2^ (cm hour^1^)	Enhancement factor (EF)
+ PS1	Solution in distilled water	6.322 (± 0.013)	1.000
PS2	Solution in IPB pH 7.4	4.458^s^ (± 0.015)	0.705
PS6	Solution in ethanolic IPB pH 7.4 (1:9)	6.490^ns^ (±0.019)	1.022

* Results are a mean of triplicate observations. SEM values are given in parentheses

+ PS1 was considered as the control study. EF = K_p_ of test solution / K_p_ of control

s Significant at *P < 0.05*

ns Not significant at *P > 0.05*. Drug Concentration = 100 *μ*g/ml

**Table 3:** Influence of various penetration enhancers on the permeability of LHCl

Study code	Penetration enhancer	*Permeability coefficient K_p_ ×10^2^(cm hr^1^)	Enhancement factor (E.F.)
PS1	Distilled water Control-1	6.322 (0.013)	1.00
PS6	Ethanolic IPB Control-2	6.490 (0.019)	1.00
PS7	Turpentine (5%)	7.450 (0.021)	1.15
PS4	DMSO (5%)	7.491 (0.032)	1.185
PS8	Pine oil (5%)	7.018 (0.036)	1.08
PS9	Menthol (5%)	7.183 (0.044)	1.11
PS5	2- Pyrrolidone (5%)	6.844 (0.052)	1.08
PS3	DMF (5%)	6.422 (0.071)	1.01

* Results are a mean of triplicate observations. SEM values are given in parenthesis. Control 1 was used for solution codes PS3, PS4, and PS5. Control 2 was used for solution codes PS7, PS8, and PS9.Drug Concentration = 100 *μ*g/ml

The effect of an increase in the concentration of the drug in the donor phase (from 100 to 200 *μ*g/ml) was also studied in both distilled water and the isotonic phosphate buffer of pH 7.4. In both cases it was found that an increase in drug concentration resulted in an increase in the permeability coefficient [Table 4]. In another study, an increase in the permeability coefficient of the drug was seen, when the concentration of DMSO was increased from 5 to 7.5 to 10% [Table 5].

**Table 4:** Effect of the quantity of drug concentration in donor cell on skin permeation of LHCl (n = 3)

Study code	Drug concentration (μg/ml)	+ Permeability coefficient K_p_ × 10^2^ cm/hr^1^	Enhancement factor (E.F.)
PS1 (D/W*)	100	6.322 (± 0.013)	1.00
PS10 (D/W)	200	7.367 (±0.016)	1.16
PS2 (IPB)	100	4.458 (± 0.015)	1.00
PS11 (IPB)	200	5.537 (±0.011)	1.24

+ Results are the mean of triplicate observations. SEM values are given in parenthesis

* Distilled water. PS1 was control for PS 10. PS2 was control for PS 11

**Table 5:** Influence of concentration of penetration enhancer on the skin permeability of LHCl

Study code	Concentration of enhancer (%)	*Permeability coefficient K_p_ × 10^2^ cm/hr^1^	Enhancement factor. (E.F.)
PS4	5	7.491 (0.032)	1.00
PS12	7.5	8.010 (0.045)	1.07
PS13	10	9.292 (0.052)	1.240

* Results are the mean of triplicate observations. SEM values are given in parenthesis. Drug Concentration = 100 *μ*/ml

## Conclusion

On the basis of these preliminary skin permeation studies we conclude that labetalol hydrochloride could be delivered through a cutaneous route, with DMSO (5 – 10%) as the penetration facilitator.
